# The association between elder abuse and childhood adversity: A study of older adults in Malaysia

**DOI:** 10.1371/journal.pone.0254717

**Published:** 2021-07-22

**Authors:** Mohammad Asyraf, Michael P. Dunne, Noran N. Hairi, Farizah Mohd Hairi, Noraliza Radzali, Choo Wan Yuen

**Affiliations:** 1 Centre for Epidemiology and Evidence-Based Practice, Department of Social and Preventive Medicine, Faculty of Medicine, University of Malaya, Kuala Lumpur, Malaysia; 2 Institute for Community Health Research, Hue University, Hue, Vietnam; 3 Australian Centre for Health Law Research, Queensland University of Technology, Brisbane, Australia; 4 Kuala Pilah District Health Office, Ministry of Health, Kuala Pilah, Malaysia; Harvard Medical School, UNITED STATES

## Abstract

**Objectives:**

Childhood adversity has been linked with later victimization of young and middle-aged adults, but few studies have shown persistence of this effect among elders, especially outside of North America. This research examined the association between adverse childhood experiences (ACEs) and elder abuse among older adults aged 60 years and over in Malaysia.

**Design:**

Cross sectional data were collected via face-to-face interview from June to August 2019.

**Setting:**

Eight government community health clinics in Kuala Pilah, a district in Negeri Sembilan state approximately 100km from Malaysian capital city Kuala Lumpur.

**Participants:**

Older adults aged 60 years and above (N = 1984; Mean age 69.2, range 60–93 years) attending all eight government health clinics in the district were recruited for a face-to-face interview about health and well-being.

**Measurement:**

The Adverse Childhood Experience International Questionnaire (ACE-IQ) and the Revised Conflict Tactics Scale (CTS) were utilized to estimate childhood adversity and elder abuse respectively.

**Results:**

Multiple logistic regression analysis revealed a significant relationship between the number of cumulative ACEs and elder abuse. Compared to older adults with no self-reported adversity, those reporting three ACEs (OR 2.67, 95% CI 1.84,3.87) or four or more ACEs (OR 1.7, 95% CI 1.16, 2.48) had higher risk of any elder abuse occurrence since age 60 years. The effect was most prominent for financial and psychological elder abuse. The associations persisted in multivariate logistic regression models after adjusting for sociodemographic and health factors.

**Conclusion:**

Early life adversities were significantly associated with victimization of older adults. Social and emotional support to address elder abuse should recognize that, for some men and women, there is a possibility that vulnerability to maltreatment persisted throughout their life course.

## Introduction

Adverse childhood experiences (ACE) can have profound effects on individuals of all ages [1–3]. Multiple studies have demonstrated that a history of adverse experiences during childhood and adolescence is linked with risky health behaviors [4–6] and many mental and physical health problems [3, 7, 8] well into adulthood [9, 10].

Early adversity can trigger a cascade of social and economic problems including low educational attainment, higher risk of unemployment, and poorer household income [11]. Adults with multiple ACEs tend to have higher healthcare utilization compared to those with no ACE [12]. When the total cost of healthcare, welfare services, and loss of productivity over the life course is considered, the economic burden amounts to many billions of US dollars annually [12, 13]. Across the East Asia and Pacific region, the health consequences of ACEs are estimated to cost up to 2% of gross domestic product [14]. It has recently been estimated that a 10% reduction in ACEs prevalence in Europe could result in annual savings of USD105 billion or 3 million Disability-Adjusted Life-Years (DALY) [12].

Another pernicious effect of adversity is that it can lead to re-victimization in later life. A systematic review of ACE studies found that people with four or more ACEs were 7.5 times (95% CI 5.60–10.08) more likely than those with no ACE to experience violent victimization as an adult [2]. While this observation is important, more research is needed to demonstrate generalizability from that meta-analysis; as the data specifically on ACE and re-victimization came from just a few high income countries, including four studies from the UK and two from the US, and none from low or middle income countries.

A further limitation is that many of the studies have assessed re-victimization occurrence in late adolescence and early to middle adult year only [2, 15] and there is scarce information on whether such associations extend to older adults. Victimization of older adults, also known as elder abuse, is a significant public health concern [16] as the global older adults population is expected to reach 2 billion by 2050 [17].

To date, most ACE-related studies of re-victimization have focused on the long-term effects of abusive acts, especially sexual and physical abuse [18–20]. Fewer studies have examined multiple types of victimization that are often reported by older people [21], including emotional, physical, sexual, and financial abuse [22]. Wang and Dong [23] found significant correlations between several types of child maltreatment and elder abuse, though the researchers acknowledged that their research captured a limited range of types of abuse, and called for further research to include broader measurement across both child maltreatment and elder abuse domains.

This study aimed to examine whether there is a significant association between ACE and elder abuse among older adults in Malaysia. It is among the first to examine this problem in Asia.

## Methods

We conducted a cross sectional, community clinic-based survey of the health and well-being of older adults in Kuala Pilah district, Malaysia from June to August 2019. Kuala Pilah is one of seven districts in the state of Negeri Sembilan, and is about 100km from Malaysia’s capital Kuala Lumpur. This district has the largest older adult population in the state, and has the highest dependency ratio of almost 50% [24].

Using census data obtained from the Kuala Pilah Health District Office, a proportional–to-size sampling calculation was done to determine the minimum number of participants needed from each of the eight government health clinics in the Kuala Pilah district to achieve a minimum target of 2000 participants. All Malaysian older adults aged 60 years and above attending the primary care clinics during the survey period were consecutively sampled until the target number of participants for each particular clinic was reached. Those eligible were approached by our team members and given detailed information about the research before being invited to participate in the survey. Those who agreed to participate were asked to sign a consent form, and all respondents were assured that they could withdraw from the study at any time without affecting the care normally received at the clinic. The questionnaire was administered via one-to-one interview by trained interviewers (all health professionals employed outside of the clinics). Interviews were conducted in a private setting within the clinics, ensuring the respondents complete privacy from the other patients or clinic staffs. The usual interview duration was between 20 to 30 minutes. The total number of older adults interviewed was 2044.

### Ethics

The study protocols and procedures were approved by the University of Malaya Research Ethics Committee (Approval Reference Number UM.TNC2/UMREC-486) and The National Medical Research Register (Approval Reference Number NMRR-19-624-45761(IIR).

## Measures

### Adverse childhood experiences

The ACE International Questionnaire (ACE-IQ) was designed by the World Health Organization to measure ACE in any country. There are 29 items in the questionnaire [25]. This questionnaire was translated and validated among Malaysian older adults in the first phase of the current study and was found to have good internal consistency (Chronbach’s Alpha = 0.701), good content validity as verified by local experts (Item Content Validity Index I-CVI>0.8), and acceptable convergent and discriminant validity.

The number of cumulative ACE was generated for each respondent using a method shown in S1 Table “Calculating the ACE score from ACE-IQ". If the respondent answered in the affirmative (whether an event occurred once, a few times, or many times) then that response was scored as 1. Total scores ranged from 0–13. This is the ACE (binary) scoring method for individuals and reflects their accumulation of ACEs.

The various types of childhood adversity are grouped into 13 categories: emotional abuse; physical abuse; sexual abuse; violence against household members; living with household members who were substance abusers; living with household members who were mentally ill or suicidal; living with household members who were imprisoned; growing up with one or no parents, parental separation or divorce during childhood; emotional neglect; physical neglect; bullying; exposure to community violence; and exposure to collective violence.

### Elder abuse

The key dependent variable in this study was elder abuse. The operational definition was based on the National Elder Abuse and Neglect study from Ireland [26]; which defined elder abuse as ‘a single, or repeated act, or lack of appropriate action, occurring within any relationship where there is an expectation of trust which causes harm or distress to an older person”. The survey questionnaire was adapted from the modified Conflict Tactic Scales (CTS) [26, 27], with minor revision. In contrast to the Irish study’s measurement of elder abuse that referred to experiences during the previous 12 months, this study took into account any abuse experienced by a respondent at any time after he or she turned 60 years old. This approach to estimating prevalence of elder abuse by accounting for any abusive experiences since age 60 years has been adopted in many studies [28]. There were 27 items in total, assessing four subtypes of elder abuse: physical, psychological, financial, and sexual abuse. If the respondent answered “yes” for any of the questions in a subtype, he or she was considered to have experienced that type of elder abuse.

### Cognitive status

The cognitive status of respondents was screened using the Elderly Cognitive Assessment Questionnaire (ECAQ). The tool assesses cognitive impairment among older adults in relation to long term memory, short term recall, and orientation [29]. The Malay version of the ECAQ has been validated for the local population [30]. Out of total score of 10, a score of 7–10 indicates normal cognitive function, 5–6 signals borderline cognitive impairment, and 0–4 indicates probable cognitive impairment.

### Covariates

Covariates for the analysis included sociodemographic information such as age, gender, ethnicity, marital status, education level, household income, employment status, living arrangement, and household income (Bottom 40% less than RM4000/USD938 monthly, Middle 40% RM4000-RM8000(USD938-USD1876), and Top 20% more than RM8000(USD1876) [31]. Sociodemographic factors has been shown to be significant determinants of ACE [32–34] and elder abuse [26, 35, 36] including a recent study of Malaysian community dwelling older adults [37].

On the basis of literature documenting associations between ACEs, elder abuse, and physical and mental health conditions, a number of health variables were included in our model as potential covariates. This included i) **Self-rated health**. Two questions where the respondents rated their recent health status as extremely good, very good, good, fair, or poor. This measure is considered to be a highly valuable indicator of general health status and is a strong predictor of mortality [38]. Self-rated health has been shown to attenuate the effect of ACE and poor outcome in later life [39, 40]. ii) **Chronic diseases**. Respondents were given a checklist of common non-communicable diseases (NCDs) such as hypertension, diabetes, cancer, heart failure, stroke, arthritis, asthma, epilepsy, visual and hearing problems. People with multiple ACEs have high odds of chronic diseases [2]; furthermore prescence of NCDs typically included as a confounding factor in various studies on elder abuse [36]. iii) **Frailty**. This was measured with the FRAIL questionnaire, a 5-item scale measuring five essential components of frailty; namely fatigue, resistance, ambulation, illness, and loss of weight [41]. Two studies conducted in Mexico and Brazil have shown that frail elders were more than twice as likely to experience elder abuse compared to those who were not frail [42, 43]. iv) **Depression**: This was measured using the Geriatric Depression Scale (Short form: GDS-15). This questionnaire has been tested and extensively used with older adults [44] and is found to be reliable and have good discriminatory properties in detecting cases and non-cases of depression among Malaysian older adults [45]. Previous systematic reviews have shown that people reporting ACE have up to five times higher odds of having depression compared to those without ACE [2, 46]. Depression is included as a confounding factor in our model as it also has been shown in longitudinal studies that elder abuse can lead to depression, and conversely, depression can also be a risk factor for elder abuse [47].

v) **Resilience**. The Brief Resilience Scale (BRS) consists of six items and is scored from zero to six. According to Smith, Dalen [48], a score of 1.00–2.99 indicates low resilience, 3.00–4.30 Normal resilience, and 4.31–500 indicates high resilience. Resilience has been cited as potential protective factor against both ACE [49, 50] and elder abuse [51, 52]. vi) **Social Support**: The interview included a brief form (11-item) of the Duke Social Support Index (DSSI) [53]. It has been validated among older adults in Malaysia [54]. A nationwide population-based survey among Malaysian older adults has shown that poorer social support resulted in higher odds of experiencing elder abuse [37].

#### Statistical analyses

The data were cleaned and checked for consistency, error, and outliers. All identified duplicates were removed. Any missing data were cross-checked with the original paper questionnaire and interviewer and revised in cases of obvious error. For example, a small number of participants had missing data on their age. To rectify this, particular interview records were cross-checked with consent forms which contained the respondent’s identity number (MyKad) and the respondent’s age was then calculated using the MyKad number. Overall, missing data in our study were minimal due to our face-to-face interview method. Out of 2044 older adults interviewed, 60 participants with ECAQ score of 4 or less (regarded as having significant cognitive impairment) were excluded from the final analysis (n = 1984). Data analysis were done using SPSS software version 25 [55].

Multiple logistic regression analyses were conducted to examine the associations between ACE and elder abuse as well as the covariates. Four models were utilized: Model 1 was the unadjusted model, Model 2 adjusted for sociodemographic factors namely age, sex, marital status, ethnicity, education level, income and living arrangement, Model 3 adjusted for sociodemographic factors included in Model 2 as well as other potential covariates, namely depression, self-rated health, frailty, and presence of chronic diseases, and finally Model 4 adjusted for all factors in Model 3 plus supportive factors (specifically resilience and social support). ACE were scored using the binary method (yes/no) and the number of cumulative ACE was the sum across the 13 ACE categories.

## Results

### Characteristics of study respondents

The final number of respondents was 1,984 and all were Malaysian older adults aged 60 years and over (mean = 69.2, SD = 6.8). Demographic features and self-reported health variables are shown in Table 1.

**Table 1:** Basic characteristics of study respondents.

Variable	All n (%)	ACE n (%)	No-ACE n (%)	*P*
**Age**				
60–69	1162 (58.6)	580 (56.4)	582 (61.0)	0.003*
70–79	625 (31.5)	324 (31.5)	301 (31.5)	
>80	196 (9.9)	124 (12.1)	72 (7.5)	
**Sex**				
Men	898 (45.3)	493 (48.0)	405 (42.4)	0.012*
Women	1086 (54.7)	535 (52.0)	551 (57.6)	
**Marital Status**				
Never Married	77 (3.9)	47 (4.6)	30 (3.2)	0.157
Divorced/Widowed	530 (26.8)	263 (25.7)	267 (28.0)	
Married	1371 (69.3)	715 (69.7)	656 (68.8)	
**Ethnicity**				
Non-Malay	356 (17.9)	191 (18.6)	165 (17.3)	0.447
Malay	1628 (82.1)	837 (81.4)	791 (82.7)	
**Education Level**				
No formal education	122 (6.2)	75 (7.4)	47 (4.9)	0.031*
Formal education	1852 (93.8)	945 (92.6)	907 (95.1)	
**Monthly Household Income (RM)**				
B40 (<RM3,999/USD938)	1912 (96.3)	994 (96.7)	918 (96.0)	0.634
M40 (RM4,000–7,999/USD939-1875)	61 (3.1)	28 (2.7)	33 (3.5)	
T20 (>RM8,000/USD1876)	11 (0.6)	6 (0.6)	5 (0.5)	
**Paid Employment**				
No	1706 (88.6)	877 (88.9)	829 (88.3)	0.694
Yes	220 (11.4)	110 (11.1)	110 (11.7)	
**Living Arrangement**				
Living Alone	205 (10.4)	105 (10.2)	100 (10.5)	0.883
Living with Others (spouse, children, others)	1776 (89.6)	921 (89.8)	855 (89.5)	
**Chronic Diseases**				
No	123 (6.2)	69 (6.7)	54 (5.6)	0.140
One	452 (22.8)	217 (21.1)	235 (24.6)	
Multiple	1409 (71.0)	742 (72.2)	667 (69.8)	
**Self-Rated Health**				
Poor	170 (8.6)	111 (10.8)	59 (6.2)	<0.001*
Good	1814 (91.4)	917 (89.2)	897 (93.8)	
**Depression**				
No	1797 (90.6)	909 (88.4)	888 (92.9)	0.001*
Yes	187 (9.4)	119 (11.6)	68 (7.1)	
**Frailty**				
Frail	487 (24.5)	271 (26.4)	216 (22.6)	0.054
Robust (Robust & Pre-Frail)	1497 (75.5)	757 (73.6)	740 (77.4)	
**Resilience**				
Low	153(7.7)	96 (9.4)	57 (6.0)	0.004*
Normal	1502(75.9)	750(73.1)	752(78.9)	
High	324 (16.4)	180(17.5)	144(15.1)	
**Social Support**^1^	29(4.5)	29.34(4.4)	29.44(4.5)	0.665

^1^ Mean (SD)

* p<0.05. p value:each sociodemographic factor vs ACE(chi-square test)

The proportion of women was marginally higher than men (54.7%, n = 1086). The ethnic composition of the sample was similar to the district population; most respondents were ethnic Malays (82.1%) and the remainders were ethnic Chinese and Indian Malaysians. The overall response rate was 92%.

The prevalence of ACE by types and cumulative number, and the prevalence of elder abuse among older adults according to types are shown in Table 2. The prevalence of ACE in our study is in line with existing ACE literature. A large US-based study involving 248,934 participants showed that 61.55% had at least 1 ACE (against ours 63.8%) and 24.64% reported 3 or more ACE (higher than ours 14.6%) [56].

**Table 2:** Prevalence of ACE and elder abuse among older adults according to subtypes.

ACE Subtypes	Overall Prevalence	Men	Women	p-value
	n (%)	n (%)	n (%)	
Any ACE	1028 (51.8)	493 (54.9)	535 (49.3)	0.012*
Physical Abuse	257 (13.1)	152 (17.1)	105 (9.8)	<0.001*
Emotional Abuse	215 (10.9)	122 (13.6)	93 (8.6)	<0.001*
Sexual Abuse	42 (2.1)	15 (1.7)	27 (2.5)	0.273
Physical neglect	471 (24.0)	230 (25.8)	241 (22.4)	0.080
Emotional neglect	125 (6.3)	55 (6.2)	70 (6.5)	0.853
Bullying	117 (5.9)	76 (8.5)	41 (3.8)	<0.001*
Alcohol and/or drug abuser in the household	24 (1.2)	12 (1.3)	12 (1.1)	0.683
Incarcerated household member	14 (0.7)	7 (0.8)	7 (0.6)	0.791
Someone chronically depressed, mentally ill, institutionalized or suicidal	15 (0.8)	7 (0.8)	8 (0.7)	0.999
Household member treated violently	137 (6.8)	63 (6.7)	74 (6.8)	0.929
One or no parents, parental separation or divorce	482 (24.4)	213 (23.8)	269 (25.0)	0.563
Community violence	404 (20.4)	250 (27.9)	154 (14.2)	<0.001*
Collective violence	92 (4.6)	44 (4.9)	48 (4.4)	0.608
**Cumulative ACE**				
**0**	615 (31.0)	222 (24.7)	393 (36.2)	<0.001
**1**	678 (34.2)	304 (33.9)	374 (34.4)	
**2**	353 (17.8)	192 (21.4)	161 (14.8)	
**3**	160 (8.1)	92 (10.2)	68 (6.3)	
**≥4**	178 (9.0)	88 (9.8)	90 (8.3)	
**Types of Elder Abuse**				
Any Abuse	466 (23.5)	236 (26.3)	230 (21.2)	0.008*
Physical Abuse	13 (0.7)	5 (0.6)	8 (0.7)	0.053
Psychological Abuse	126 (6.4)	59 (6.6)	67 (6.2)	<0.001*
Sexual Abuse	7 (0.4)	1 (0.1)	6 (0.6)	0.024*
Financial Abuse	400 (20.2)	207 (23.1)	193 (17.8)	<0.001*

Our study’s prevalence of zero ACE was 36.2%, and the prevalence of respondents reporting 4 or more ACE was 8.3%. These estimates are within the ranges found in a systematic review of 37 ACE studies globally which reported that the prevalence of zero ACE ranged from 12% to 67% and prevalence of at least four ACE ranged from 1% to 38% [2]. Growing up with one or no parent was the most common ACE (prevalence 24.4%, n = 482) followed by physical neglect (24%, n = 471) and exposure to community violence (20.4%, n = 404). Gender subgroup analysis showed that ACEs were reported significantly more often among men compared to women (54.9% vs 49.3% respectively). Men were observed to have experienced significantly more cumulative ACE compared to women. In terms of elder abuse, financial abuse was the most prevalent at 20.2% (n = 400) while sexual abuse was reported least often at 0.4% (n = 7).

Table 3 shows binary associations between cumulative ACEs and specific types of elder abuse, with no ACE as the reference group. It can be seen that generally, the higher the number of cumulative ACE, the stronger the association with elder abuse. When the ACE score was 1, the slightly higher odds of having any type of elder abuse did not reach statistical significance. Having 2 ACEs was significantly associated with elder financial abuse. Those with ACE score of 3 were 2.67 times more likely to experience elder abuse of any type, and this was largely related to elevated risk of financial and psychological elder abuse.

**Table 3:** Relationship between cumulative adverse childhood experiences and elder abuse subtypes.

No. of ACE	Any Abuse		Financial Abuse		Psychological Abuse		Physical Abuse		Sexual Abuse	
	OR (95%CI)	p-value	OR (95% CI)	p-value	OR (95% CI)	p-value	OR (95% CI)	p-value	OR (95% CI)	p-value
1	1.01 (0.77, 1.33)	0.926	1.05 (0.78, 1.39)	0.767	0.79 (0.48, 1.31)	0.362	4.56 (0.53, 39.15)	0.166	1.61 (0.17, 15.46)	0.679
2	1.35 (0.99, 1.84)	0.060	1.40 (1.01, 1.94)	0.043*	1.19 (0.69, 2.06)	0.530	3.50 (0.32, 38.72)	0.307	0.39 (0.01, 26.69)	0.661
3	2.67 (1.84, 3.87)	<0.001*	2.54 (1.73, 3.75)	<0.001*	2.30 (1.28, 4.16)	0.006*	3.86 (0.24, 62.08)	0.340	3.29 (0.23, 47.42)	0.382
≥4	1.70 (1.16, 2.47)	0.006*	1.45 (0.97, 2.18)	0.072	2.16 (1.21, 3.86)	0.009*	14.12 (1.57, 127.10)	0.018*	8.94 (1.05, 75,79)	0.045*

* p<0.05

Note: OR presented with no ACE as reference group

Those with ACE score of 4 or more had significantly higher rates of three of the four subtypes of elder abuse (physical abuse, psychological abuse, and sexual abuse). The confidence intervals for the ORs for physical and sexual abuse were very wide as the numbers of cases were quite low (n = 13, 0.7% for physical abuse; n = 7, 0.4% for sexual abuse).

Outcomes from multivariable logistic regression analyses are summarized in Table 4 for unadjusted and adjusted models. The unadjusted model (Model 1) shows a trend for a dose-response relationship between cumulative ACE and elder abuse. Adjusting for sociodemographic factors in Model 2 produced a similar graded relationship. In Model 3, which adjusted for sociodemographic factors included in Model 2 plus self-rated recent health, depression, frailty, and presence of chronic diseases, the effect was significant at higher levels of ACE (3, or 4 and above) but not at the lower levels of ACE (1 or 2). Model 4 added two putative supportive factors, (resilience and social support) to Model 3, but the reduction in the respective odds ratio was minimal.

**Table 4:** Adjusted multivariable relationship between cumulative adverse childhood experiences and elder abuse.

ACE		Elder Abuse		
	Model 1	Model 2	Model 3	Model 4
	Unadjusted Odds Ratio (95% CI)	Adjusted Odds Ratio (95% CI)	Adjusted Odds Ratio (95% CI)	Adjusted Odds Ratio (95% CI)
ACE				
No	1	1	1	1
Yes	1.19 (0.97, 1.47)	1.23 (0.98, 1.54)	1.18 (0.95, 1.46)	1.17 (0.94, 1.46)
0	1	1	1	1
1	1.01 (0.77, 1.33)	0.96 (0.72, 1.27)	0.96 (0.72, 1.27)	0.95 (0.72, 1.27)
2	1.35 (0.99, 1.84)	1.32 (0.96, 1.83)	1.27 (0.92, 1.77)	1.25 (0.90, 1.74)
3	2.67 (1.84, 3.87)*	2.45 (1.66, 3.62)*	2.33 (1.57, 3.48)*	2.32 (1.56, 3.47)*
≥4	1.70 (1.16, 2.47)*	1.73 (1.17, 2.56)*	1.71 (1.15, 2.56)*	1.71 (1.14, 2.56)*

Model 1 –unadjusted model

Model 2 –adjusted for sociodemographic factors namely age, sex, marital status, ethnicity, education level, income and living arrangement.

Model 3 –adjusted for all sociodemographic factors in Model 2, plus relevant health covariates namely self-rated health, depression, frailty and chronic diseases.

Model 4 –adjusted for all factors in Model 3 plus potential supportive factors namely resilience and social support.

## Discussion

To our knowledge, this is the first research to examine associations between adverse childhood experiences and elder abuse in Asia and in any middle-income country. The primary finding is that older adults who had experienced multiple childhood adversities were significantly more likely to report having experienced elder abuse since the age of 60 years. This pattern remained after adjustment for various sociodemographic and health-related confounding factors.

This study confirms recent research among elder white Americans [57] and members of a USA Chinese community [23] that show significant associations between childhood maltreatment and risk of victimization as older adults. The latter study identified three subtypes of child maltreatment (physical, psychological, and sexual) as predictors of elder abuse; Kong and Easton [57] included questions on child abuse and five other ACE subtypes namely neglect, family dysfunction, parental divorce, witnessing domestic violence, and growing up with a household member who had a substance misuse problem. Our study extends this work by incorporating thirteen types of childhood adversities using the ACE International Questionnaire [25]. Together with the North American research, this Malaysian study indicates that the experience of multiple adversities during childhood predisposes people to abuse when they are elders.

A strength of this study is that elder abuse was measured using an extensive list of 27 items modified from the Conflict Tactic Scale (CTS) that enable estimation of four types of elder abuse: physical, psychological, sexual, and financial abuse. This adds to contemporary research by Easton and Kong [58] which used aggregate measures to categorize elder abuse victimization.

The association between ACEs and elder abuse could arise from a complex interaction of socio-biological factors. Neurologically, it has been postulated that adverse childhood experiences can alter brain structures involved in the perception of, and response to, fear-inducing stimuli, and altering brain regions that are essential for learning and memory [59, 60]. Severe adversity can disrupt higher order brain development and may have direct and indirect impact on key aspects of adult life, and this may account for higher susceptibility to victimization during adult years, including for people in the final decades of life.

Recently, Easton and Kong [58] suggested that cumulative advantage/disadvantage theory may explain the links between childhood adversity and health problems in later life. In a longitudinal survey of almost 6000 older adults in the USA, they argued that childhood adversities undermine multiple health-related factors in middle adulthood, which may increase the affected individuals’ susceptibility to victimization later in the life course. Their study found significant indirect effects of childhood adversities on elder abuse victimization through two domains of midlife health factors, namely physical health problems and depressive symptoms. Childhood adversity has been shown to compromise these two factors in adolescent and people in early to middle adult years, perhaps best referred to as wear and tear of the body due to chronic toxic stress. This strain also leads to harmful compensatory mechanisms such as smoking, substance misuse and unhealthy diet. Accumulated disadvantage across the life course induces disabilities and chronic mental and psychosocial conditions which, in turn, may increase the vulnerability of older people to psychological and financial maltreatment and other types of abuse.

Although this study in Malaysia has some strengths, the findings should be interpreted with caution due to methodological limitations. All ACE and elder abuse measurement was based on self-report from respondents during face-to-face interviews, thereby introducing risk of recall limitation and social desirability-response bias. The adverse experiences may be underreported due to the sensitive nature of the subject. For example, it is often considered taboo among conservative Malaysian older adults to disclose unwanted sexual experiences regardless of what age they occurred. Another limitation is that the sampling at only government clinics in a rural area of Kuala Pilah may limit the generalizability of our findings to the wider population. The sampling method effectively excludes older adults in the population who do not attend these clinics. This includes those who are very healthy and visit clinics rarely, and also those who may be unwell but avoid health care. Of special concern is that our sample did not include people who are immobile residents of aged care facilities, who may be at risk of elder abuse. Additionally, our selection criteria effectively excluded those who can afford to have their primary health care in private practices, which represent the more affluent subgroup of older adults in the district. Lastly, the cross-sectional nature of the survey, while practical and cost-effective, will not allow us to infer a causal relationship between ACE and elder abuse.

## Conclusion

Using a large, representative sample of community-dwelling older adults in a state of Malaysia, this is the first research in Asia and in any middle-income country to demonstrate that the odds of experiencing elder abuse increased with exposure to ACE in a dose-response way.

The findings shed light on how ACEs can affect individuals throughout the life course, particularly by identifying ACE as one of potential predictors of elder abuse. This study contributes to the emerging base of evidence to support efforts to screen individuals for childhood adversities, especially multiple types of ACE, and intervene via health and social services to reduce risk of re-victimization. Such interventions include higher investment in public health programs to promote routine clinical interviewing for childhood adversity among individuals presenting with victimization-related health problems, regardless of their age. This is important, because it could prevent re-victimization.

Further research should be designed to elucidate the intermediary pathways between ACE and elder abuse. This should yield theoretically informative data, and most importantly, could identify ways in which health systems and social services can intervene to minimise the harm.

## Supporting information

S1 Table Calculating ACE score from ACE-IQ. (DOCX)
