# Supplementary material for: The association between elder abuse and childhood adversity: A study of older adults in Malaysia
Source: PLoS One. 2021 Jul 22;16(7):e0254717. doi: 10.1371/journal.pone.0254717 (PMC8297753; doi:10.1371/journal.pone.0254717)
Supplement: S1 Table — (DOCX) [file pone.0254717.s001.docx]

**S1 Table**: **Calculating the ACE score from the ACE-IQ**

| **Category** | **Question** |
| --- | --- |
| **Physical abuse** | Did a parent, guardian or other household member spank, slap, kick, punch or beat you up?  OR  Did a parent, guardian or other household member hit or cut you with an object, such as a stick (or cane), bottle, club, knife, whip etc? |
| **Emotional abuse** | Did a parent, guardian or other household member yell, scream or swear at you, insult or humiliate you?  OR  Did a parent, guardian or other household member threaten to, or actually, abandon you or throw you out of the house? |
| **Contact sexual abuse** | Did someone touch or fondle you in a sexual way when you did not want them to?  OR  Did someone make you touch their body in a sexual way when you did not want them to?  OR  Did someone attempt oral, anal, or vaginal intercourse with you when you did not want them to?  OR  Did someone actually have oral, anal, or vaginal intercourse with you when you did not want them to? |
| **Alcohol and/or drug abuser in the household** | Did you live with a household member who was a problem drinker or alcoholic, or misused street or prescription drugs? |
| **Incarcerated household member** | Did you live with a household member who was ever sent to jail or prison? |
| **Someone chronically depressed, mentally ill, institutionalized or**  **suicidal** | Did you live with a household member who was depressed, mentally ill or suicidal? |
| **Mother Household member treated violently** | Did you see or hear a parent or household member in your home being yelled at, screamed at, sworn at, insulted or humiliated?  OR  Did you see or hear a parent or household member in your home being slapped, kicked, punched or beaten up?  OR  Did you see or hear a parent or household member in your home being |
| **One or no parents, parental separation or divorce** | Were your parents ever separated or divorced? OR  Did your mother, father or guardian die? |
| **Emotional neglect** | Did your parents/guardians understand your problems and worries? OR  Did your parents/guardians **really** know what you were doing with your free time when you were not at school or work? |
| **Physical neglect** | Did your parents/guardians **not** give you enough food even when they could easily have done so?  OR  Were your parents/guardians too drunk or intoxicated by drugs to take care of you?  OR  Did your parents/guardians **not** send you to school even when it was available? |
| **Bullying** | Were you bullied? |
| **Community violence** | Did you see or hear someone being beaten up in real life? OR  Did you see or hear someone being stabbed or shot in real life? OR  Did you see or hear someone being threatened with a knife or gun in real life? |
| **Collective violence** | Were you forced to go and live in another place due to any of these events?  OR  Did you experience the deliberate destruction of your home due to any of these events?  OR  Were you beaten up by soldiers, police, militia, or gangs? OR  Was a family member or friend killed or beaten up by soldiers, police, militia, or gangs? |
